# Melatonin and Cisplatin-Associated Skeletal Muscle Wasting and Atrophy: Mechanistic Rationale, Current Evidence, and Research Gaps

**DOI:** 10.3390/muscles5040076

**Published:** 2026-10-16

**Authors:** Mohamed A. A. Hassan, Mahmoud Abd-Elnaeim, Enas A. Abd-Elhafez, Ramy K. A. Sayed

**Affiliations:** 1 Department of Anatomy and Embryology, Faculty of Veterinary Medicine, Sohag University, Sohag 82524, Egypt; m.abdalhamed@vet.sohag.edu.eg; 2 Department of Anatomy and Embryology, Faculty of Veterinary Medicine, Assiut University, Assiut 71526, Egypt; naeim61@vet.au.edu.eg; 3 Department of Cell and Tissues, Faculty of Veterinary Medicine, Assiut University, Assiut 71526, Egypt; enas.mahmoud@vet.au.edu.eg

**Keywords:** cisplatin, melatonin, skeletal muscle atrophy, chemotherapy-induced myotoxicity, mitochondrial dysfunction, oxidative stress, ubiquitin–proteasome system, apoptosis

## Abstract

Cisplatin causes skeletal muscle wasting in tumor-free rodents, reducing muscle mass, myofiber size and, where measured, strength. This critical narrative review appraises that phenotype and its mechanistic basis. Tumor-bearing models show that the loss also occurs under tumor burden, but most cannot separate drug from tumor effects. Melatonin influences mitochondrial, redox, regenerative, and structural outcomes in skeletal muscle under non-cisplatin conditions. Direct melatonin–cisplatin evidence is limited to pretreatment studies in C2C12 cells from one research group. No in vivo melatonin-plus-cisplatin skeletal-muscle study and no clinical study of melatonin against cisplatin-associated muscle loss were identified in searches through 22 September 2026. Biological plausibility is supported, but protection has not been demonstrated. A controlled tumor-free study would provide a more interpretable test of whether melatonin protects skeletal muscle during cisplatin exposure. Translational validation would then require a separate tumor-bearing study testing whether protection persists under tumor burden and whether cisplatin’s antitumor activity is preserved.

## 1. Introduction

Cisplatin entered clinical oncology in the late 1970s and, more than four decades later, platinum compounds remain among the most widely used anticancer drugs, including in the era of targeted therapy and immunotherapy [1]. Continued use means that off-target tissue effects remain clinically relevant; cisplatin-associated hepatotoxicity has also been demonstrated experimentally in rats [2]. Skeletal muscle has not traditionally been regarded as a primary target organ of cisplatin toxicity [3], yet a substantial experimental literature now documents muscle loss after cisplatin administration in animals carrying no tumor.

In tumor-free mice and rats, cisplatin reduces whole-muscle mass, decreases myofiber size, and impairs grip strength, endurance, and contractile force [4,5,6,7,8]. These are models of drug exposure in otherwise healthy animals. They are not models of cancer cachexia or of age-related sarcopenia, and describing them in those terms merges distinct pathophysiologies; the distinction is maintained throughout.

Muscle status is not merely descriptive in platinum-treated patients. In a retrospective cohort of patients with locally advanced head and neck squamous cell carcinoma treated with primary chemoradiotherapy and high-dose cisplatin, low pretreatment skeletal muscle mass was associated with a higher frequency of cisplatin dose-limiting toxicity than normal muscle mass [9]. That association concerns muscle mass measured before treatment and does not by itself establish that treatment-associated muscle loss subsequently worsens tolerance. It does establish that skeletal muscle is a clinically consequential compartment during cisplatin therapy, which is reason enough to ask whether the muscle injury reproduced in animals can be limited.

Melatonin is a plausible candidate for that question. It acts through MT1 and MT2 receptor signaling and through receptor-independent redox and mitochondrial actions [10]. Additionally, the experimental work in skeletal muscle has shown that melatonin can attenuate mitochondrial depolarization and permeability-transition-pore opening in muscle cells under oxidative challenge [11], improve selected contractile measures in dystrophic mice [12], and modify regeneration-associated outcomes after chemical muscle injury [13]. Several of the processes it engages overlap with processes disturbed in cisplatin-exposed muscle.

The evidential position is nonetheless asymmetric, and that asymmetry defines this review. Cisplatin-associated muscle injury is supported by a broad experimental base spanning structure, function, and several molecular domains. Melatonin has muscle-relevant biological actions demonstrated in models unrelated to chemotherapy. Evidence placing the two together in muscle-lineage tissue consists of two publication records from a single research group in C2C12 cells [14,15]. No in vivo skeletal-muscle study and no clinical study addressing melatonin against cisplatin-associated muscle loss were identified in the targeted search underlying this review.

This critical narrative review therefore has three aims: (i) to characterize the cisplatin skeletal-muscle phenotype and its mechanistic basis at the level the primary experiments actually support; (ii) to appraise the evidence using an author-defined organizational framework rather than a formal grading system: Class A, direct melatonin + cisplatin skeletal-muscle/myogenic-cell evidence; Class B, cisplatin skeletal-muscle evidence without melatonin; Class C, melatonin skeletal-muscle evidence from non-cisplatin models; Class D, melatonin + cisplatin evidence from non-muscle organs; and Class E, broader mechanistic/translational context; and (iii) to specify what a defensible in vivo test of the melatonin hypothesis would require.

### Literature Search and Evidence Classification

This article is a critical narrative review and was not conducted according to a registered or predefined systematic- or scoping-review protocol; no quantitative data were pooled or meta-analyzed. The initial targeted literature search was conducted through 9 August 2026, primarily using PubMed/MEDLINE, and was supplemented by general web searches and screening of the reference lists of relevant primary studies and review articles. An updated search was performed on 22 September 2026, followed by targeted verification of selected records against their primary sources when clarification of study characteristics, experimental context, or potentially overlapping publications was required. Scopus, Embase, and Web of Science were not included in the documented search process. Complete historical search strings were not retained, and contemporaneous language-filter settings were not recorded. Accordingly, the review does not assert a predefined language restriction, and these limitations are reported to avoid retrospectively reconstructing search parameters that were not documented at the time of searching.

The literature search, screening of identified records, and study selection were undertaken collaboratively by the first author and another human researcher. The process was not designed or documented as formal independent duplicate screening, and no predefined disagreement-adjudication procedure was documented. Primary experimental and clinical studies were considered when they addressed the relevant intervention, exposure, skeletal-muscle outcome, or mechanistic question and could be assigned to one of the evidence classes defined in the review. Review articles were used for background context and to identify relevant primary studies rather than as primary evidence. Full texts were consulted whenever accessible, whereas records for which only an abstract was available are explicitly identified as such.

To preserve distinctions between biologically and experimentally different forms of evidence, studies were recorded separately according to experimental format (in vivo animal, ex vivo, or cell-based), tumor status (tumor-free or tumor-bearing), and skeletal-muscle outcome. A study was classified as providing tumor-bearing evidence only when tumor-bearing animals received cisplatin and a skeletal-muscle endpoint was evaluated in the corresponding treatment arm. Tumor-free cisplatin studies were retained as mechanistic or proof-of-concept evidence but were not considered models of cancer cachexia. Potential overlap between publications was considered conservatively. Shared authorship, laboratory affiliation, or similar experimental protocols were not, by themselves, interpreted as evidence of shared animals or samples. Conversely, when the independence of experimental cohorts could not be established from the available information, records were neither counted as confirmed independent replications nor automatically merged. This approach was intended to avoid both unsupported assumptions of duplication and overcounting of potentially related evidence.

The A–E evidence classes used in this review are author-defined organizational categories based on intervention or exposure, experimental model, and tissue context. They are intended to structure the narrative synthesis and distinguish different types of evidence; they do not represent evidence-quality grades, formal risk-of-bias assessments, or a hierarchical scoring system.

The studies summarized in the manuscript and tables should therefore be regarded as representative of the evidence identified through the search and verification procedures described above rather than as an exhaustive systematic-review inventory. Accordingly, statements that a particular study type was “not identified” refer specifically to the searches and source-verification procedures undertaken for this review and should not be interpreted as evidence that no such study exists in the broader literature.

## 2. Cisplatin-Associated Skeletal Muscle Wasting: Experimental Landscape and Phenotype

### 2.1. Tumor-Free Models: Phenotype and Experimental Context

Most of what is known about cisplatin and skeletal muscle comes from short-term rodent experiments in tumor-free animals, in which the drug is given to healthy mice or rats, and muscle is collected within days of the final dose. A smaller group superimposes cisplatin on an implanted tumor. Zhang et al. [16] treated Lewis lung carcinoma-bearing mice and reported reductions in gastrocnemius mass and fiber cross-sectional area (CSA) that linalool attenuated dose-dependently, but that design has no healthy cisplatin-only arm, so drug and tumor contributions cannot be separated within it. Tumor-bearing work is therefore treated as a contrast to the tumor-free literature rather than an extension of it.

Within the tumor-free literature, mice predominate, most often male C57BL/6J [3,4,5,8,17,18,19], while rats include Wistar strains [6,20,21] and Lister-hooded rats [22]. Dosing is heterogeneous. Muscle selection varies across quadriceps, gastrocnemius, tibialis anterior, extensor digitorum longus (EDL), and triceps. Male animals dominate; female cohorts are less common [21,23], and only Huot et al. [7] stratified by sex and age, so both remain thinly covered.

Cisplatin also reduces food intake and body weight, raising the question of how much muscle loss is secondary to reduced intake. The most informative designs include dietary-restriction (DR) comparators matched to treated-animal intake. Cisplatin reduced hindlimb and quadriceps mass, quadriceps fiber diameter, and induced atrogenes and FOXO3 beyond matched restriction [4], and reduced quadriceps mass, grip strength, and treadmill endurance relative to both saline and DR groups [8]. These comparisons show an atrophic component not fully explained by reduced intake, but they do not exclude every systemic cisplatin effect. Pair-feeding abolished the muscle lipogenic-enzyme differences reported by Garcia et al. [24], indicating that not every molecular change is drug-specific. Recovery data show persistent muscle loss after food intake and body weight recovery [25], and continuing mass loss after dosing stops [19].

Endpoints differ in what they can support. Whole-muscle wet weight is organ-level evidence, not morphometry. Quantified myofiber diameter or CSA sits at the morphometric level and is available in a minority of studies [4,5,18,19,22,25,26], with Damrauer et al. [27] reporting gastrocnemius fiber diameter at limited quantitative depth. Several influential mechanistic and functional papers contain no morphometric endpoint [3,7,8,17,20,28], and qualitative hematoxylin–eosin inspection, as in Aremu et al. [21], does not substitute for quantified morphometry.

The resulting phenotype is consistent at the organ level, with reduced muscle weight across quadriceps, gastrocnemius, tibialis anterior, EDL, and triceps in mice and rats. Where morphometry was performed, myofiber size also fell, including reduced quadriceps fiber diameter versus saline and DR comparators [4] and reduced mean fiber CSA with a leftward size-distribution shift [19]. Fiber-type-resolved data are scarce and model-specific; Brierley et al. [22] reported selective IIx/IIb hypotrophy in rat EDL, while Sekine et al. [25] reported fiber-type-resolved CSA changes in gastrocnemius. Functional impairment was documented as reduced grip strength [3,5,6,8], treadmill endurance [8], wire-hang performance [21], and, in Huot et al., reduced plantarflexion torque across wild-type strata and stratum-specific ex vivo EDL force changes, not detected in young females [7]. These results are not pooled across age or sex strata, and locomotor activity is treated as complementary rather than equivalent to direct muscle function [25]. Representative studies are summarized in Table 1.

### 2.2. Tumor-Bearing Models: What They Add and What They Cannot Separate

Cisplatin is given to patients who have tumors. A tumor-free model cannot reproduce tumor-derived catabolic signaling or tumor-associated anorexia, and it cannot capture the possibility that reducing tumor burden itself changes the course of muscle loss. Tumor-free models therefore answer a narrower question, namely what cisplatin does to skeletal muscle in an otherwise healthy host, and they remain the mechanistic basis of this review. Tumor-bearing models answer a different question: how muscle responds when cisplatin acts on a host that already carries a tumor.

In the evidence assembled for this review, eight studies gave cisplatin to tumor-bearing rodents and measured at least one skeletal-muscle endpoint [16,27,29,30,31,32,33,34]. Seven are supported by full-text or frozen-project extraction; the eighth is indexed-abstract only, so its dose, sex, muscle identity, sample size, and morphometry are not quantified here [34]. Two additional studies reported only organ-level muscle weights in tumor-bearing cisplatin arms [35,36]. Drug-versus-tumor separation depends on inclusion of a tumor-free cisplatin comparator. Damrauer et al. [27] included tumor-free and C26-bearing mice; muscle wasting persisted under cisplatin, while MuRF1 induction differed with tumor status and NF-κB was activated in the tumor-free cisplatin arm. Cabrera et al. [30] used a factorial cancer × chemotherapy design and found no significant interaction for gastrocnemius mass, although interactions were reported for several molecular endpoints; this is not evidence of additivity or synergy.

The remaining tumor-bearing studies lacked a tumor-free cisplatin comparator. Four closely similar LLC studies from one research group used male C57BL/6 mice and 4 mg/kg cisplatin and reported quantified fiber CSA and forelimb grip strength [16,29,31,32]. These designs provide a cisplatin contrast only within tumor burden; their similarity narrows methodological diversity but does not imply shared animals or samples. An orthotopic lung-cancer xenograft study likewise had tumors in every cisplatin-treated group and reported muscle wet weight and image-sampled fiber size, but no functional endpoint [33].

Across most of this literature, tumor and cisplatin effects remain incompletely separable. Cisplatin also reduced tumor burden in several models, so muscle outcomes reflect tumor progression, drug toxicity, and treatment response together. These short-schedule rodent experiments are not treated as models of cancer cachexia as a clinical syndrome. They show that cisplatin-associated muscle loss also occurs under tumor burden, but they do not replace tumor-free models as the basis for mechanistic inference. Representative tumor-bearing studies are listed in Table 1.

**Table 1:** Representative experimental evidence for cisplatin-associated skeletal muscle wasting in tumor-free and tumor-bearing models. Compact representative set (21 studies; tumor-free studies first, followed by tumor-bearing studies); not a systematic or pooled inventory. Wet/whole-muscle mass is organ-level evidence, not morphometry; qualitative H&E is not quantified morphometry; both are labeled as such below. “None” indicates the endpoint is absent from the study.

Study	Model/Species/Sex	Tumor Status	Cisplatin Exposure	Dietary-Control Design	Muscle(s)	Mass/Morphometry	Functional Endpoint	Principal Mechanistic Contribution	Key Limitation
Sakai et al. [4]	Mouse C57BL/6J, male, 8–9 wk	Tumor-free	1 or 3 mg/kg i.p., daily, Days 0–3 (4 doses; 12 mg/kg cumulative at 3 mg/kg)	Pair-fed DR	Hindlimb/quadriceps	Hindlimb/quadriceps mass ↓ (organ-level); quadriceps fiber diameter ↓ vs. saline and DR (quantified morphometry)	None	Atrogin-1/MuRF1/FOXO3 ↑ and Akt/FOXO phosphorylation ↓ beyond DR	DR does not exclude all systemic effects
Conte et al. [6]	Rat Wistar, male adult	Tumor-free	1 mg/kg i.p., Days 1–3 (3 doses; 3 mg/kg cumulative)	None; food recorded	EDL (principal); TA; forelimb	EDL weight ↓ (organ-level); EDL CSA ↓ (quantified)	Grip ↓; ex vivo EDL electrophysiology/Ca^2+^ handling abnormal	MuRF1 ↑; Ca^2+^-handling genes altered; GHS partly improved morphometric, functional and Ca^2+^ endpoints	Lc3/Bnip3 unchanged at collection; no flux inference or DR comparator
Sirago et al. [28]	Rat, strain NR, male adult	Tumor-free	1 mg/kg i.p., Days 1–3 (3 mg/kg cumulative)	None	TA	TA weight ratio ↓ (organ-level only)	None	PGC-1α/NRF1/TFAM and mtDNA ↓; redox imbalance; steady-state autophagy markers altered	No morphometry/function; no flux or DR comparator
Sakai et al. [17]	Mouse C57BL/6J, male, 8–9 wk	Tumor-free	3 mg/kg i.p., daily ×4 (12 mg/kg cumulative)	Matched DR	Quadriceps	Quadriceps mass (mg/g, denominator unstated)—organ-level only	None	Ubiquitin genes/protein and ubiquitinated-protein pool ↑ beyond DR; clearance discrimination in C2C12 only	No morphometry/function; cell result not transferred
Sakai et al. [18]	Mouse C57BL/6J, male, 8–9 wk	Tumor-free	3 mg/kg i.p., daily ×4 (12 mg/kg cumulative)	Matched DR	Quadriceps	Quadriceps mass ↓, partially preserved by mecasermin; fiber diameter ↓, partially restored (quantified)	None	IGF-1/PI3K/Akt suppression; mecasermin restored signaling and partly suppressed atrogenes/restored diameter	Partial attenuation only; no function
Wu et al. [23]	Mouse C57BL/6, female, 6 wk	Tumor-free	2 mg/kg i.p., weekly ×5 (10 mg/kg cumulative)	None stated; food NR	Bilateral gastrocnemius	None (no discrete mass/morphometry endpoint; TEM/TUNEL/ROS only)	None	Apoptotic and ferroptosis-associated panel; pathway dissection in C2C12 only	No quantified atrophy endpoint; ferroptosis inferred from a combined panel, not established in vivo
Sato et al. [8]	Mouse C57BL/6J, male, 8–9 wk	Tumor-free	3 mg/kg i.p., daily ×4 (12 mg/kg cumulative)	Matched DR	Quadriceps	Quadriceps mass (mg/g) ↓ vs. saline and DR; no histology anywhere (confirmed absence)	Forelimb/four-paw grip ↓; treadmill endurance ↓ vs. saline and DR	ATP/OXPHOS/mtDNA-nDNA/PGC-1α ↓ vs. both comparators	No morphometry; PGC-1α specificity finding, not demonstrated causality
Sekine et al. [25]	Mouse C57BL/6J, male, 8 wk	Tumor-free, recovery design	3 mg/kg/day i.p., Days 0–3 (12 mg/kg cumulative)	None; food/BW measured	Gastrocnemius, plantaris	GC/plantaris mass loss persists to Day 14 despite systemic recovery of food intake/BW; fiber-type-resolved CSA (red/white GC)—quantified	Grip ↓; locomotor activity ↓ (behavioral/complementary, not equivalent to a direct muscle-function endpoint); HET did not significantly restore grip	MyoD suppressed; slow-twitch miRNA changes; HET accelerated selected recovery	Recovery design; slow-fiber findings muscle-specific
Aremu et al. [21]	Rat Wistar, female, 200–250 g	Tumor-free	3 mg/kg/day i.p., daily ×5, cisplatin from Day 4 of NAC treatment (15 mg/kg cumulative)	None; BW measured, food not locked	Triceps	Triceps wet weight ↓ (organ-level only); qualitative H&E only—not quantified morphometry	Wire-hang and grip ↓	Redox/TFAM/miRNA changes; compensatory PGC-1α ↑; N-acetylcysteine improved selected redox/miRNA endpoints	No CSA; mtDNA/nDNA not restored; dose wording conflict
Brierley et al. [22]	Rat Lister-hooded, male, 200–225 g	Tumor-free	Single 6 mg/kg i.p.	None; food/BW/activity monitored	EDL	EDL mass ↓ (organ-level); fiber-type-specific CSA ↓—selective IIx/IIb hypotrophy (quantified)	Locomotor activity only (not counted as a direct muscle-function endpoint)	Steady-state LC3/p62 dysregulation; no flux assay; cannabigerol partly restored Akt/S6, not SGK1	Single-dose acute model; no flux/cell arm
Liu et al. [26]	Mouse WT C57BL/6 and PD-1 KO, male, 8 wk	Tumor-free; genetic-intervention model	2.5 mg/kg i.p., Days 1–5 and 20–25 (25 mg/kg cumulative)	None; food not claimed	Quadriceps, gastrocnemius, soleus, TA	Four muscle weights ↓ (organ-level); TA CSA/fiber diameter ↓ (quantified)	None	Atrogene/inflammatory/oxidative/ferroptosis-autophagy panels; PD-1 limits phenotype	Global KO; muscle-intrinsic dependence and flux/ferroptosis not established
Chen et al. [19]	Mouse C57BL/6J, male, 7 wk	Tumor-free (time-course, macrophage-depletion and ginkgetin arms)	3 mg/kg/day i.p., Days 0–4 or Days 3–8 by arm (15 or 18 mg/kg cumulative; schedules not merged)	None; food not measured	Gastrocnemius	GC mass/ratio progressively ↓ (organ-level); mean CSA and size-distribution ↓ (quantified); attenuated by clodronate depletion and by ginkgetin	None	Macrophage/M1 and inflammatory markers; clodronate/ginkgetin attenuated mass/CSA loss	Systemic depletion supports macrophage contribution, not cGAS–STING dependence in vivo; docking computational only; no direct C2C12 protection
Sakai et al. [5]	Mouse C57BL/6J, male, 8–9 wk	Tumor-free	3 mg/kg i.p., Days 0–3 (12 mg/kg cumulative)	Matched DR	Quadriceps	Quadriceps mass partially preserved by bortezomib; fiber diameter partially preserved (quantified, ≥80 fibers/animal)	Forelimb strength NOT restored by bortezomib	20S proteasome induction; bortezomib did not suppress MuRF1/atrogin-1 or correct body-weight/food-intake loss	Partial structural preservation; no functional restoration
Huot et al. [7]	Mouse C57BL/6J WT and muscle-specific PGC1α transgenic; 2/18 mo, male/female; four strata	Tumor-free; genetic-intervention model (no tumor inoculation—authors’ own stated limitation)	2.5 mg/kg i.p.; 9 injections over a 14 d period (22.5 mg/kg cumulative)	None reported	Gastrocnemius, TA, quadriceps (mass); EDL (ex vivo force); triceps surae (electrophysiology)	IBW-normalized mass loss in every stratum, though not in every muscle (young-female quadriceps not reported as reduced); organ-level ratio only—no CSA/morphometric endpoint of any kind; PGC1α overexpression gave stratum-specific, partial, non-generalizable mass protection	Plantarflexion torque reduced in all WT + cisplatin strata; EDL specific-force deficits stratum-specific and not detected in young females. PGC1α overexpression improved torque across strata and selected ex vivo force/MUNE endpoints, with partial and stratum-specific effects	Muscle-specific PGC1α overexpression: partial, stratum-specific mass/motor-unit protection, strongest across endpoints in aged males; mitochondrial-protein loss stratum-specific	Four strata not pooled/generalized; no genotype × treatment interaction test
Seo et al. [20]	Rat Wistar, male, ~7–8 wk	Tumor-free	0.1 or 0.5 mg/kg i.p., daily ×14 (1.4 or 7.0 mg/kg cumulative)	None; ad libitum food	Gastrocnemius	Absolute GC weight ↓ at 0.5 mg/kg (organ-level); GC/BW ratio unchanged—a null result	None	RCR ↓ at 0.5 mg/kg (cross-sectional, not causal); steady-state autophagy markers ↑, p62 unchanged; no flux claim	No lysosomal-inhibitor arm; null GC/BW ratio; low-dose schedule differs from dominant protocol
Nanri et al. [3]	Mouse C57BL/6J, male, 8–9 wk	Tumor-free (animal arm); C2C12 myotube arm recorded separately	3 mg/kg i.p., daily ×4 (12 mg/kg cumulative)	DR only in separate SUnSET experiment; not in TUDCA arm	Quadriceps	Quadriceps mass normalized to body weight ↓, partially attenuated by TUDCA; absolute mass not displayed; no morphometry—histology not performed (authors’ explicit limitation)	Grip strength (% change Day 0 → 4) ↓, partially improved by TUDCA; TUDCA alone had no effect on strength or body weight	ER-stress markers ↑, p-p70S6K ↓, MuRF1/atrogin-1 ↑; TUDCA attenuated markers and mass/grip loss; PERK–ATF4–Ddit4 chain C2C12-only	Mass is a ratio; no morphometry; cell-only causal chain not transferred; SUnSET arm lacked TUDCA; cohort independence unresolved
Damrauer et al. [27]	Mouse CD2F1, sex NR	Mixed: tumor-free cisplatin and C26-bearing ± cisplatin	5 mg/kg (route unresolved), Days 7, 10, 14 (~15 mg/kg cumulative)	None; food NR	Gastrocnemius	GC mass ↓ (organ-level); H&E fiber-diameter measurement ↓—limited quantitative morphometry	None	Tumor-free arm: NF-κB activation without MuRF1 induction; MuRF1 induction tumor-dependent; wasting persisted despite tumor reduction	Route unreported; small endpoint-specific n; fiber CSA not reported; tumor/drug effects partly separable; 2018 reprint not independent
Cabrera et al. [30]	Mouse BALB/c, male and female; sexes analyzed separately	C26-bearing factorial + healthy cisplatin comparator	2.25 mg/kg i.p. per injection, 4 injections in two cycles (days 12, 15, 19, 22; 9 mg/kg cumulative)	None; no DR/pair-fed comparator	Gastrocnemius	GC mass (organ-level): cancer main effect in both sexes and chemotherapy main effect in females only; no significant cancer × chemotherapy interaction; no fiber CSA	None	Cancer × chemotherapy interactions for selected molecular endpoints; no interaction for gastrocnemius mass	No morphometry/function or pair-feeding; endpoint-specific interactions do not establish additivity, synergy or a distinct mechanism
Zhang et al. [16]	Mouse C57BL/6, male, 5 wk at treatment	Tumor-bearing animal (LLC); no healthy cisplatin-only arm	4 mg/kg i.p., every 3 d, Days 7–18 (4 doses; 16 mg/kg cumulative, figure-derived)	None; food intake/tumor volume monitored daily	Gastrocnemius (morphometry + molecular); TA (mass)	GC/TA mass ↓ (organ-level); GC mean CSA ↓ with leftward size-distribution shift (quantified)	Forelimb grip strength ↓, Day 18	Atrogene/ubiquitin ↑; IGF-1/Akt/FoxO dependence shown only in C2C12	No healthy cisplatin-only arm; pathway dependence cell-only
Chi et al. [29]	Mouse C57BL/6, male, 5 wk	Tumor-bearing animal (LLC); tumor-only arm; no healthy cisplatin-only arm	4 mg/kg i.p., every 2 days ×4 (16 mg/kg cumulative)	None; food intake measured	Gastrocnemius (morphometry + molecular); TA (mass)	GC/TA mass ↓ (organ-level); GC CSA ↓ and fiber-type distribution altered (quantified); attenuated by silibinin	Forelimb grip strength ↓; improved by silibinin	Atrophy/myogenic/redox markers; ERK/FoxO and JNK/FoxO involvement shown only in C2C12	No healthy cisplatin-only arm; pathway dependence cell-only; sample-size reporting inconsistent
Kim et al. [33]	Mouse BALB/c nu/nu, male, 5 wk	Tumor-bearing orthotopic A549; no healthy cisplatin-only arm	8 mg/kg i.p., once weekly (administration count not stated; cumulative exposure not calculable)	None; body weight and feed rate measured	Quadriceps femoris, gastrocnemius	Wet weight ↓ by approximately 30% (organ-level); fiber size from three H&E images per group (image-sampled, not per-animal)	None	Myostatin and IL-6/TNF-α ↑, attenuated by PLAG; tumor burden assessed	Immunodeficient xenograft; n inconsistencies; abstract “up to 70%” unsupported by Results

BW = body weight; CSA = cross-sectional area; DR = dietary restriction (matched or pair-fed comparator); EDL = extensor digitorum longus; GC = gastrocnemius; GHS = growth hormone secretagogue(s); GPX4 = glutathione peroxidase 4; GSH = glutathione; HET = hochuekkito; IBW = initial body weight; IGF-1 = insulin-like growth factor 1; i.p. = intraperitoneal; LLC = Lewis lung carcinoma; MDA = malondialdehyde; MUNE = motor-unit number estimation; NAC = N-acetylcysteine; NR = not reported; PGC-1α = peroxisome proliferator-activated receptor-gamma coactivator 1-alpha; RCR = respiratory control ratio; SUnSET = surface sensing of translation (puromycin-incorporation assay); TA = tibialis anterior; TUDCA = tauroursodeoxycholic acid; WT = wild type. ↑ = increased; ↓ = decreased, as reported in the cited study; the arrows alone do not indicate statistical significance.

## 3. Mechanistic Convergence in Cisplatin-Associated Muscle Wasting

### 3.1. Proteostasis and Anabolic Suppression

Induction of muscle-specific ubiquitin ligases is recurrent in cisplatin-exposed muscle: MuRF1 and atrogin-1/MAFbx rose in mouse quadriceps/gastrocnemius [4,18,19,26,37] and MuRF1 in rat EDL [6]. D-methionine attenuated FOXO1/MAFbx induction and improved fiber diameter/CSA, although MuRF1 did not differ significantly [38]. DR-controlled designs further showed atrogene/FOXO3 induction beyond matched restriction [4]. Ubiquitin supply and ubiquitinated proteins also increased beyond DR [17]. In C2C12 myotubes, inhibitor experiments supported proteasomal rather than autophagic clearance, but this pathway distinction was not established in vivo.

Bortezomib preserved quadriceps mass and fiber diameter in part but did not restore forelimb strength or suppress MuRF1/atrogin-1 [5]. This supports a proteasomal contribution to structural loss without establishing it as the sole determinant. Damrauer et al. [27] further showed gastrocnemius loss with NF-κB activation but without MuRF1 induction in tumor-free cisplatin-treated mice, indicating that MuRF1 induction was not required in that model.

The anabolic side shows a parallel pattern. Cisplatin reduced muscle IGF-1/PI3K/Akt signaling and increased MuRF1/atrogin-1, while exogenous IGF-1 restored signaling, partially suppressed atrogenes, and partially restored fiber diameter [18]. This is partial attenuation, not normalization. Reduced Igf1 transcript and altered Akt/S6 signaling were also reported in other tumor-free models [22,37]. In the tumor-bearing linalool study, IGF-1/Akt/FoxO dependence was tested only in C2C12 cells, so it cannot be extended to the animal findings [16]. Capsaicin and silibinin provided additional intervention evidence for structural/functional attenuation, but their mechanistic dissection was cell-restricted [29,39].

Akt-mediated phosphorylation ordinarily restrains FOXO transcriptional activity, providing a mechanistic link between reduced anabolic signaling and the atrogene program. In cisplatin-exposed mouse quadriceps, lower Akt/FOXO3a phosphorylation accompanied increased FOXO3, MuRF1 and atrogin-1/MAFbx expression beyond matched dietary restriction [4]. These associations do not establish FOXO-dependent causality in vivo.

### 3.2. Endoplasmic Reticulum Stress and Translational Suppression

Cisplatin engages the unfolded-protein response in muscle. In quadriceps, ER-stress markers increased, p70S6K phosphorylation fell, and MuRF1/atrogin-1 rose; tauroursodeoxycholic acid (TUDCA) attenuated these changes and the losses in body-weight-normalized quadriceps mass and grip strength, without correcting body-weight loss [3]. Translational suppression was demonstrated separately by reduced puromycin incorporation measured by SUnSET, including relative to DR, but no TUDCA arm tested rescue of protein synthesis. The in vivo relationship between ER stress and translational suppression therefore remains association-level. The PERK–ATF4–Ddit4/REDD1 chain was established in C2C12 cells and is not transferred to animals. A multi-drug screen provided convergent ER-stress marker changes without intervention; because cohort independence is unresolved, it is not treated as an independent replication [37].

### 3.3. Mitochondrial Dysfunction and Redox Imbalance

Mitochondrial readouts are affected across species and muscles. In DR-controlled mice, quadriceps ATP, OXPHOS transcripts, mtDNA/nDNA, and PGC-1α fell with reduced grip and endurance [8]. PGC-1α was the only candidate regulator falling versus both comparators, a specificity finding rather than a causal chain. In rat tibialis anterior, PGC-1α/NRF-1/TFAM and mitochondrial/redox measures were altered [28]. Ex vivo respirometry in rat gastrocnemius showed a lower respiratory control ratio at the higher dose, but relationships with molecular markers were cross-sectional [20].

Muscle-specific PGC1α overexpression protected plantarflexion torque across the four age/sex strata, but mass protection was partial in some strata, absent in young females and indeterminate in aged females; motor-unit effects also differed by stratum [7]. Mitochondrial protein loss was stratum-specific. No endpoint-specific genotype-by-treatment interaction was reported, and no stratum is generalized to another.

Redox disturbance accompanied these changes, with oxidative-damage markers in rat tibialis anterior and elevated ROS/malondialdehyde with depleted glutathione in mouse gastrocnemius [23,28]. N-acetylcysteine improved redox markers and selected miRNAs but did not restore the mtDNA/nDNA ratio [21]. PGC-1α increased rather than decreased in that model, underscoring that its direction is not uniform across species, muscles, and schedules.

### 3.4. Regulated Cell Death and Autophagy-Related Signaling

Apoptotic signaling is supported by cleaved caspase-3/PARP, cytochrome c release, TUNEL positivity, and abnormal mitochondrial ultrastructure in mouse gastrocnemius [23]. The same study reported a ferroptosis-associated panel and used a pan-caspase inhibitor/ferrostatin-1 in C2C12 cells. Ferroptosis therefore remains an inference from a combined panel, not a single marker or established in vivo causal mechanism; the same caution applies to PD-1-deficient mice [26]. In C2C12 myotubes, p53 inhibition reduced ROS, mitochondrial dysfunction, and Bax/Bcl-2/atrogene changes but did not rescue myosin-heavy-chain loss, supporting only a partial, cell-restricted contribution [40].

Autophagy-related readouts are similarly bounded. Steady-state LC3-II/LC3-I, Beclin 1, and BNIP3 increased in rat gastrocnemius at the higher dose while p62 was unchanged, with no lysosomal-inhibitor arm [20]. LC3/p62 dysregulation was also reported in rat EDL without dynamic flux measurement [22], while another rat model showed no significant EDL LC3/Bnip3 transcript change [6]. No study established in vivo autophagic flux; steady-state markers cannot distinguish increased throughput from blocked degradation, so the direction and functional consequence remain undetermined.

Autophagic homeostasis requires effective lysosomal cargo clearance, not merely autophagosome formation. In muscle biopsies from patients with LAMP2-related Danon disease, Nascimbeni et al. associated impaired autophagy with p62-positive aggregates and myofiber atrophy [41]. This inherited myopathy illustrates the consequences of defective clearance but does not establish a comparable lysosomal defect after cisplatin exposure.

### 3.5. Inflammatory, Immune and Regenerative Responses

Macrophage infiltration and M1 polarization increased over time, with CCL5/IFN-β/IL-6 elevation; systemic clodronate depletion attenuated muscle weight/CSA loss and atrogene expression [19]. This supports a macrophage contribution but not macrophage cGAS–STING dependence in vivo, which was interrogated in RAW264.7 cells; ginkgetin had no direct protective effect on C2C12 myotubes. Global PD-1 knockout aggravated muscle loss and inflammatory/oxidative/catabolic markers, indicating a limiting role for endogenous PD-1, but muscle-intrinsic dependence cannot be inferred [26]. NF-κB activation in the tumor-free cisplatin arm of Damrauer et al. [27] was associated with this response. A DR-controlled screen found no significant TNF-α, IL-1β, or COX2 transcript changes at the examined time point [37], emphasizing readout- and time-dependence.

Regenerative capacity is less well covered. MyoD transcript remained suppressed while gastrocnemius and plantaris mass stayed reduced through day 14 despite recovery of food intake and body weight, and hochuekkito, which accelerated recovery and raised slow-twitch-associated microRNAs, did not significantly restore grip strength at the day-13 comparison [25]. Those slow-fiber observations belong to the muscles examined and are not generalized to EDL or to muscle as a whole.

Neuromuscular junction (NMJ) changes may contribute to cisplatin-associated weakness beyond reduced myofiber size. In a separate tumor-free cisplatin-treated mouse cohort, Huot et al. reported lower motor unit number estimates, reduced NMJ-associated proteins, and altered presynaptic morphology [42]. These results suggest impaired motor unit connectivity without establishing that NMJ disruption causes muscle atrophy. Nor do they demonstrate that NMJ injury explains the persistent muscle loss or delayed recovery noted above.

### 3.6. What the Mechanistic Evidence Supports

**Figure 1:**
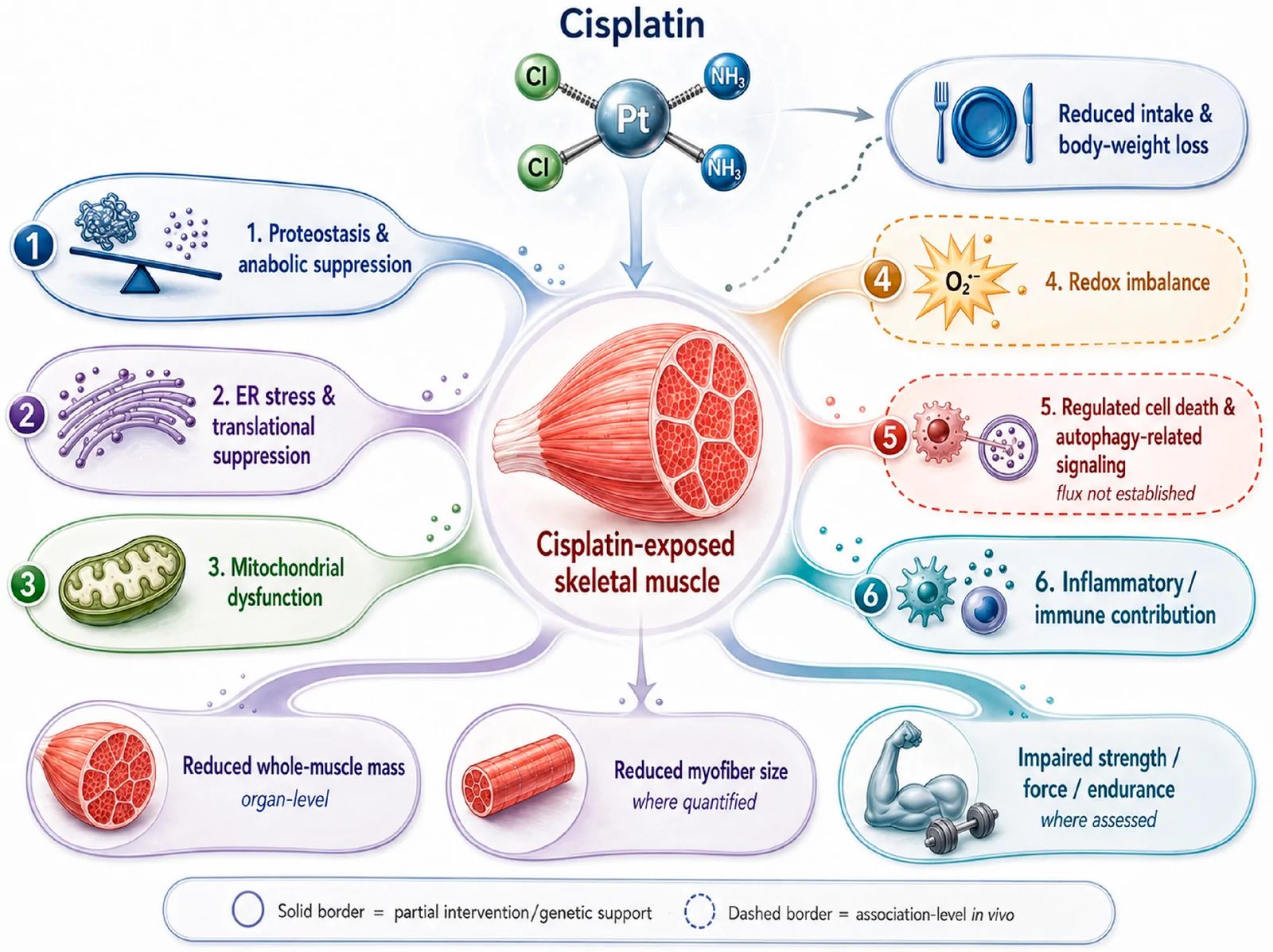
Cisplatin-associated skeletal muscle wasting: phenotype and mechanistic convergence. In tumor-free rodents, cisplatin exposure reduces food intake and body weight and perturbs six overlapping mechanistic domains in skeletal muscle: proteostasis and anabolic suppression, endoplasmic-reticulum stress and translational suppression, mitochondrial dysfunction, redox imbalance, regulated cell death and autophagy-related signaling, and inflammatory/immune contribution. Solid module borders mark domains for which endpoint-specific pharmacological or genetic intervention provides partial supporting evidence; dashed borders mark domains that remain association-level in vivo. The autophagy-related node is explicitly qualified because steady-state marker changes do not establish autophagic flux. The resulting phenotype comprises reduced whole-muscle mass, an organ-level endpoint; reduced myofiber size, reported only where morphometry was quantified; and impaired strength, force, or endurance, reported only where function was directly assessed. No single domain is depicted as sufficient to explain the phenotype, and no melatonin content is included in this figure.

No single pathway specifies the phenotype. Proteasomal, IGF-1-axis, ER-stress, mitochondrial, and macrophage-directed interventions each attenuate part of it but leave measurable deficits. Structural, functional, and molecular readouts often dissociate, and heterogeneity in dose, schedule, muscle, and collection time limits direct comparison. The evidence supports a multi-node disturbance of proteostasis, anabolic signaling, and mitochondrial function rather than a single linear pathway. Melatonin is considered because its muscle evidence intersects particularly with mitochondrial function, redox regulation, and regulated cell death; its relevance to other domains is less direct. Figure 1 summarizes this architecture.

## 4. Melatonin Biology Relevant to Skeletal Muscle

### 4.1. Mechanistic Properties Relevant to Muscle

Melatonin (N-acetyl-5-methoxytryptamine) is synthesized principally by the pineal gland from serotonin through sequential acetylation by arylalkylamine N-acetyltransferase (AANAT) and O-methylation by acetylserotonin O-methyltransferase (ASMT/HIOMT) [43]. AANAT activity is the circadian rate-limiting step, rising during the dark phase under sympathetic, cAMP/PKA-dependent control and falling with light exposure, producing the characteristic nocturnal melatonin peak [43]. Beyond this principal pineal source, melatonin synthesis and local action have also been reported in numerous extrapineal tissues, including gut, skin, and immune cells [44]; both sources are cited here as physiological background only and report no skeletal-muscle or cisplatin-specific findings. Melatonin can influence skeletal-muscle biology through receptor-dependent signaling and receptor-independent redox and mitochondrial actions [10]. These properties provide a plausible mechanistic bridge to several disturbances identified in Section 3, particularly mitochondrial dysfunction, redox imbalance and regulated cell death. The evidence reviewed here does not, however, establish that MT1- or MT2-dependent signaling mediates a protective effect of melatonin in cisplatin-exposed skeletal muscle, and receptor dependence cannot be assumed across tissues. Receptor-dependent modulation of cisplatin toxicity by melatonin has been demonstrated experimentally in a non-muscle organ, which is discussed in Section 5.3; no equivalent receptor-manipulation experiment in skeletal muscle under cisplatin exposure was identified.

Recent aging and circadian-disruption models add structural and functional endpoints. In naturally aged male C57BL/6J mice, long-term melatonin treatment was associated with higher gastrocnemius mass ratio, larger fiber CSA, and greater forelimb grip strength [45]. The study also linked these outcomes to gut-derived inflammatory and metabolic signals, but that mechanism is indirect to cisplatin and is not expanded here beyond what the design supports. In inducible muscle-specific Bmal1-knockout mice, Fernández-Martínez et al. [46] studied both sexes and included a melatonin-alone arm; melatonin improved gastrocnemius mass and fiber-size measures, treadmill-related performance, and selected mitochondrial/ultrastructural outcomes. These findings arise from a circadian-disruption model rather than toxic injury and are not transferred to cisplatin exposure.

A companion mechanistic report in the same iMS-Bmal1 model included a distinct melatonin-alone arm and added myogenic-gene, mitochondrial-dynamics, and respirometry endpoints [47]. It did not re-establish the structural or functional phenotype de novo, and its exact melatonin dose, route, timing, and duration were not independently verified in the source set used here. The two publications are therefore treated as complementary reports on the same model framework; reuse of animals, tissues, or samples is neither established nor inferred.

### 4.2. Mitochondrial and Redox Effects in Skeletal-Muscle Models

The most direct muscle-specific mechanistic evidence concerns mitochondrial responses to oxidative stress. In primary skeletal-muscle cultures prepared from C57BL/6J mice, melatonin attenuated tert-butyl-hydroperoxide-induced mitochondrial depolarization and mitochondrial permeability-transition-pore opening and maintained a more reduced intracellular environment, with corresponding protection against subsequent cell death [11]. These findings establish an experimentally supported mitochondrial/redox action of melatonin in muscle cells, but the insult was oxidative rather than chemotherapeutic.

A separate study in undifferentiated C2C12 myoblasts tested chemically distinct apoptotic triggers, including hydrogen peroxide, etoposide, and staurosporine. Melatonin pretreatment attenuated oxidative stress and apoptosis in this non-cisplatin injury model, with the authors implicating the mitochondrial pathway at least in part [48]. The records available for this review do not resolve the outcome trigger by trigger, so no uniform effect across all triggers tested is claimed here. The value of this experiment is not that it predicts a response to cisplatin, but that cytoprotection in muscle-lineage cells was demonstrated for more than one type of chemical injury. Neither study measured an in vivo myofiber-atrophy endpoint, and cell-level mitochondrial protection cannot be equated with preservation of muscle mass, fiber size, or function.

### 4.3. Muscle Structure, Regeneration and Function in Non-Cisplatin Models

Animal studies extend the melatonin evidence base into functional and structural outcomes, but in pathologies that differ substantially from chemotherapy-associated wasting. In mdx5Cv mice, a genetic model of Duchenne muscular dystrophy, melatonin treatment improved selected triceps surae contractile measures, including twitch and maximal tetanic tension, reduced plasma creatine kinase activity, and improved muscle redox status [12]. These findings demonstrate an intervention effect on dystrophic-muscle function and biochemical injury markers; they do not indicate correction of the dystrophin defect and cannot be transferred to an acquired toxic model.

In a glycerol-induced tibialis anterior injury model, combined with mechanistic experiments in C2C12 cells, melatonin treatment improved rotarod performance, muscle structural readouts, and fiber-size measures during early recovery and was linked experimentally to Pax7-dependent myogenic differentiation [13]. The animal and cell components must remain separate: the Wnt/β-catenin, miR-3475-3p, and Pax7 manipulations were used primarily to dissect differentiation signaling in C2C12 cells, whereas the mouse arm supplied injury, morphology, and performance outcomes. This study therefore provides direct evidence that melatonin can modify regeneration-related outcomes in a non-cisplatin muscle-injury model, not evidence that it prevents chemotherapy-associated atrophy.

A third context is chronic metabolic disease. In Zucker diabetic fatty rats, melatonin altered red vastus lateralis muscle structure and the expression of proteins involved in mitochondrial fission and fusion, together with changes in oxidative phenotype and in autophagy-related markers, including LC3- and p62-related readouts [49]. No dynamic flux experiment was performed, so, for the reasons set out in Section 3.4, these are treated here as changes in autophagy-associated markers, and melatonin is not described as restoring, activating, or inhibiting autophagic flux in skeletal muscle. This model also differs from both dystrophy and acute injury and should not be treated as an equivalent atrophy paradigm.

Additional non-cisplatin paradigms extend this evidence base further. In a rat crush-injury model, melatonin increased twitch and tetanic muscle force, satellite-cell number, and MT1a receptor expression while reducing leukocyte infiltration and apoptosis [50]. In aged mice, melatonin mitigated loss of muscle mass and strength, replenished the satellite-cell pool, and reduced fibrogenic conversion of satellite cells, a sarcopenia-relevant paradigm distinct from the injury models above [51]. In a cardiotoxin-injury model, melatonin treatment in vivo was associated with reduced fibro-adipogenic-progenitor senescence and improved post-injury muscle regeneration and repair-related outcomes; in isolated cells from this model, AMPK-pathway manipulation was required for melatonin’s anti-senescence effect, a dependence established only at the cell level [52]. In a frostbite-injury model, melatonin altered slow- versus fast-fiber marker composition in injured tibialis anterior in vivo, while separate C2C12 experiments showed mitochondrial bioenergetic effects sensitive to the AMPK inhibitor Compound C, a pathway dependence not extended to the animal findings [53]. Finally, in a hindlimb ischemia–reperfusion model, melatonin reduced oxidative stress and inflammatory markers and cell death in injured muscle [54]. As with the paradigms discussed above, none of these studies involved cisplatin exposure, and their heterogeneous models are not pooled into a single mechanism.

Collectively, the dystrophic, glycerol-injury, diabetic, crush-injury, sarcopenia, cardiotoxin, frostbite, ischemia–reperfusion, and chronic immobilization-stress studies show that melatonin can influence function, regeneration, morphology, and mitochondrial organization in skeletal muscle under several non-cisplatin conditions [55], while the heterogeneity of the underlying insults prevents those effects from being pooled into a single mechanism of muscle protection.

### 4.4. Proteostasis and Anabolic-Signaling Markers in Non-Cisplatin Models

Two studies address the proteostasis and anabolic-signaling domains that are prominent in cisplatin-exposed muscle (Section 3.1). In mice with cecal ligation and puncture-induced sepsis, melatonin pretreatment was reported to attenuate the reduction in tibialis anterior fiber CSA, with associated changes in MuRF1 and PI3K/Akt signaling; the animal melatonin dose is not stated in the accessible record [56]. Melatonin was given as a 7-day pretreatment before injury. Akt dependence was tested with the Akt inhibitor MK-2206 only in lipopolysaccharide-treated C2C12 myotubes and is not extended to the animals. The study measured neither muscle mass nor muscle function, and atrogin-1 was not reported. In leptin-deficient ob/ob mice, whose hindlimb muscle weight was about 27% lower than that of wild-type controls, melatonin modified markers of the ubiquitin–proteasome system, macroautophagy, chaperone-mediated autophagy and the unfolded-protein response [57]. These were steady-state measurements, so they are treated as autophagy-marker responses rather than evidence of altered autophagic flux (Section 3.4). MuRF1 was reduced rather than elevated in ob/ob muscle, the opposite direction to that reported after cisplatin. Both studies extend melatonin’s documented muscle actions to proteostasis-related signaling, but under inflammatory and metabolic insults whose catabolic programs differ from cisplatin exposure.

**Figure 2:**
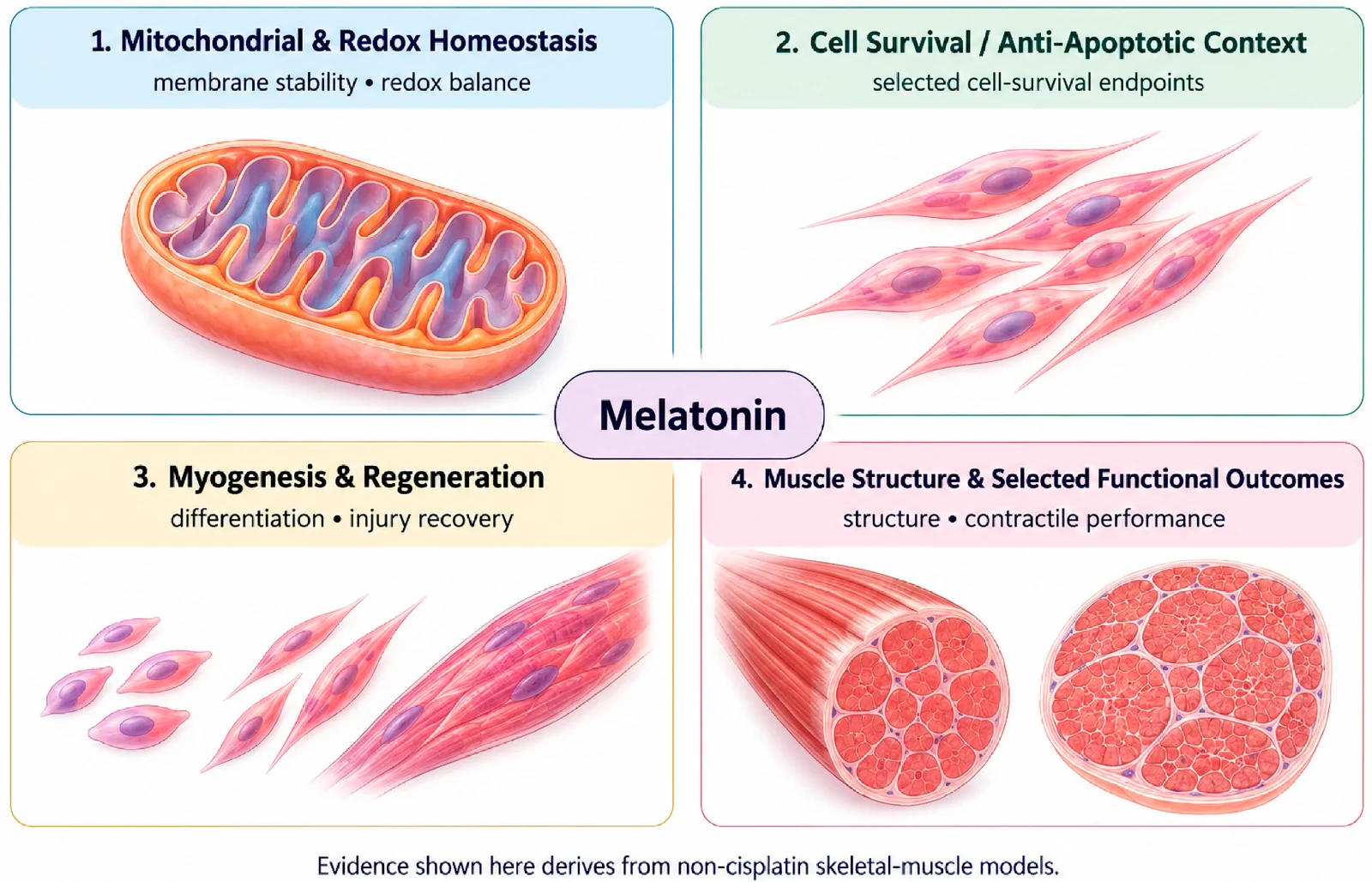
Melatonin actions relevant to skeletal muscle in non-cisplatin models. Four categories are shown: mitochondrial and redox homeostasis; cell survival; myogenesis and regeneration; and muscle structure with selected functional outcomes. The underlying studies used oxidative, chemical, crush, cardiotoxin, frostbite and ischemia–reperfusion injury; genetic dystrophy; diabetic, obese and septic mice; aging; and circadian disruption. Proteostasis-related marker changes from septic and obese mice (Section 4.4) are discussed in the text and are not shown as a separate figure element. Cell and animal findings are reported separately and are not pooled. None of these studies involved cisplatin. Direct cisplatin evidence is shown in Figure 3.

Every skeletal-muscle observation summarized in this section derives from a non-cisplatin model. The available muscle studies establish that melatonin can engage biological processes relevant to muscle maintenance, mitochondrial redox control, contractile performance, regeneration, and structural remodeling, under other forms of injury or disease. They do not establish that those effects occur in cisplatin-exposed skeletal muscle. Biological plausibility of this kind is a reason to test a hypothesis, not a substitute for testing it, and the cisplatin-specific question requires a separate appraisal of the direct cell-level evidence and of the explicitly indirect supporting literature. The principal skeletal-muscle-relevant actions of melatonin discussed in Section 4 are summarized in Figure 2.

**Figure 3:**
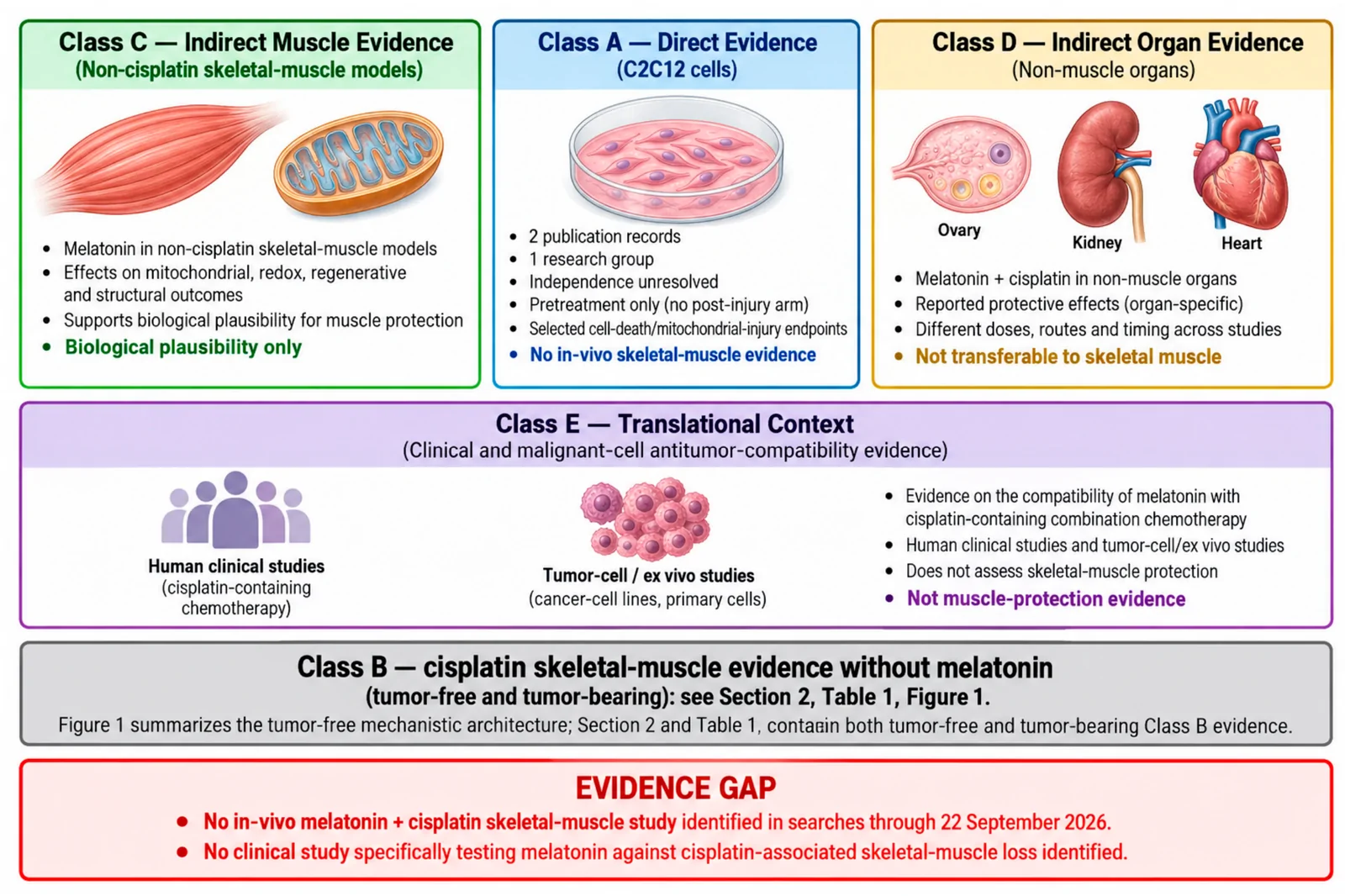
Evidence boundary for melatonin in cisplatin-associated skeletal-muscle wasting, organized by evidence class. Class A (direct) consists of two publication records from one research group in C2C12 cells, both using melatonin pretreatment. Their experimental independence is unresolved, and endpoints are limited to selected cell-death and mitochondrial-injury readouts. Class B (cisplatin skeletal-muscle evidence without melatonin, from tumor-free and tumor-bearing models) is shown as a reference bar; Figure 1 depicts the tumor-free mechanistic architecture, while Section 2 and Table 1 present both the tumor-free and the tumor-bearing Class B evidence. Classes C (melatonin in non-cisplatin muscle models), D (melatonin plus cisplatin in non-muscle organs), and E (heterogeneous translational context: clinical studies and malignant-cell antitumor-compatibility data) are indirect. The classes are organizational categories, not quality grades, and no connector implies transfer of support between them. The gap box refers to searches through 22 September 2026.

## 5. Melatonin Against Cisplatin-Associated Myotoxicity: Direct and Indirect Evidence

### 5.1. Direct Skeletal-Muscle/Myogenic-Cell Evidence

Two publication records place melatonin directly alongside cisplatin in a skeletal-muscle-lineage cell system. Both originate from the same research group, and their experimental independence is unresolved; they must not be counted as two confirmed independent experiments, and equally must not be collapsed into a proven shared dataset.

The full article exposed proliferating, undifferentiated C2C12 myoblasts to cisplatin (30 µM, 24 h) with melatonin (100 µM) applied as pretreatment 24 h before the cisplatin challenge [15]. Using transmission electron microscopy, TUNEL/confocal laser-scanning microscopy, and cardiolipin/NAO-associated mitochondrial readouts, melatonin pretreatment attenuated selected apoptotic and mitochondrial-injury endpoints associated with cisplatin exposure. A caspase-9 inhibitor was used in the same study to characterize the injury pathway; this result describes how cisplatin kills the cells, not how melatonin protects them, and should not be read as establishing melatonin’s mechanism of action. The design is a preconditioning paradigm: melatonin was applied for 24 h before cisplatin, no post-injury arm wash was included, and the available Methods do not establish whether melatonin remained in the medium during the cisplatin challenge. The study used a high in vitro melatonin concentration (100 µM), which limits direct clinical extrapolation. It is a myoblast study: it contains no muscle-mass endpoint, no myofiber measurement, no animal arm, and no autophagy assessment, and its quantitative coverage of the combined melatonin-plus-cisplatin flow-cytometry arm is not established with sufficient certainty to support strong numerical claims. Because regulated cell death is one of several convergent processes implicated in cisplatin-exposed muscle in Section 3.4, this study provides a cell-level anchor for that specific domain; it does not speak to proteostasis, anabolic signaling, or the other mechanistic domains discussed there, and its findings should not be read as evidence about the phenotype as a whole.

The second record is a supplement abstract describing C2C12 myoblasts and myotubes pretreated with melatonin before exposure to several chemical triggers, cisplatin among them, again examined by transmission electron microscopy and TUNEL/confocal laser-scanning microscopy [14]. The abstract of record states that melatonin pretreatment prevented apoptosis induced by hydrogen peroxide, cisplatin, and etoposide, whereas staurosporine-induced apoptosis was not inhibited, which the authors attributed to a mechanism of action other than an increase in free radicals. The inclusion of differentiated myotubes broadens the direct evidence beyond proliferating myoblasts, but the abstract does not separate the myoblast and myotube results and does not report cisplatin concentration, melatonin concentration, exposure duration, sample size, statistical testing or effect size. None of these details can be reconstructed from the available record. One further feature of the pair is recorded without interpretation: the two records state opposite outcomes for staurosporine [14,15], the abstract reports that staurosporine-induced apoptosis was not inhibited, while the later article’s title reports prevention of staurosporine-induced apoptosis. This divergence is noted as a provenance observation; it is not treated as evidence either that the two records rest on independent experiments or that they share a dataset.

Taken together, direct cell-level evidence of melatonin-associated attenuation of cisplatin-related apoptotic and mitochondrial injury in muscle-lineage cells exists, but it is small in scope, derives from two records from one research group with experimental independence unresolved, is restricted to selected injury endpoints, and offers no atrophy, mass, or functional measurement of any kind.

### 5.2. What Indirect Skeletal-Muscle Evidence Contributes

The non-cisplatin melatonin-in-muscle literature reviewed in Section 4 cannot substitute for direct cisplatin evidence, but it clarifies what is already known about melatonin’s action in muscle-lineage tissue and what remains untested. Mitochondrial and redox protection demonstrated in primary muscle cultures and in C2C12 myoblasts exposed to non-cisplatin oxidative or apoptotic triggers [11,48] supplies biological plausibility for the mitochondrial-protective signal reported in the direct cisplatin experiment, since both describe melatonin acting on comparable subcellular targets. The dystrophic, glycerol-injury, and diabetic-muscle intervention studies additionally show that melatonin’s actions are not confined to isolated cells and can extend to tissue-level outcomes under other forms of injury [12,13,49,50,51]. None of this literature, however, was generated under cisplatin exposure, and none provides a structural, functional, or mass endpoint under a chemotherapy insult. The indirect evidence therefore supplies a rationale for testing whether melatonin’s demonstrated cellular and tissue-level actions extend to cisplatin-exposed skeletal muscle, without supplying any evidence that such an extension actually occurs.

### 5.3. Melatonin Plus Cisplatin Evidence in Non-Muscle Organs

Two cisplatin-specific melatonin studies outside skeletal muscle provide organ-level and mechanistic context. In a mouse ovarian cisplatin model, a nonselective MT1/MT2 receptor antagonist inhibited melatonin-associated ovarian protection, whereas a selective MT2 antagonist did not, a pattern that supports involvement of the MT1 receptor in that organ [58]. This demonstrates that receptor-dependent modulation of cisplatin toxicity by melatonin is experimentally achievable and gives a template for how the question could eventually be approached in muscle, but the result was generated in ovarian tissue and must not be read as evidence of MT1 dependence in skeletal muscle, where no comparable receptor-manipulation experiment has been performed under cisplatin exposure. A second cisplatin-ovary study implicated the PTEN/AKT/FOXO3a axis in melatonin’s protective context [59]; because FOXO-family signaling also regulates the atrogene program described in Section 3.1, this result indicates pathway-level overlap with a mechanism relevant to muscle atrophy, not that the same pathway dependence has been shown to operate in cisplatin-exposed muscle.

Representative evidence from other non-muscle organs extends this picture. In the kidney, early work showed that melatonin reduced cisplatin-induced elevations in blood urea nitrogen, creatinine, and renal lipid peroxidation in mice [60], and a later study linked melatonin’s nephroprotection to activation of the Nrf2/HO-1 pathway in rats [61]. Two clinical trial publications have tested melatonin against cisplatin-induced nephrotoxicity in cancer patients. In a pilot, randomized, double-blinded, placebo-controlled trial, melatonin-treated patients had numerically fewer acute kidney injury (AKI) episodes and smaller changes in selected urinary injury biomarkers, but the study was small and did not establish a statistically significant AKI-prevention effect [62]. In a second randomized controlled trial in 66 patients, cisplatin caused urinary magnesium and potassium loss; melatonin was associated with directionally lower AKI prevalence and urinary electrolyte loss, but these differences were not statistically significant, and the authors concluded that melatonin ameliorated urinary electrolyte loss without demonstrating prevention of acute renal injury [63]. In the liver, melatonin attenuated cisplatin-associated hepatic histopathological, inflammatory (TNF-α), and biochemical (ALT, AST, LDH, albumin) abnormalities in rats [64]. In the brain, melatonin improved oxidant/antioxidant status and reduced proinflammatory cytokine elevation after cisplatin exposure in rats [65]. In the heart, melatonin attenuated cisplatin-induced cardiac troponin and LDH-1 elevation and apoptotic markers, an effect abolished by a BDNF-receptor antagonist, indicating BDNF-pathway dependence in that organ [66]. As with the ovarian findings above, none of this organ-level evidence is transferred to skeletal muscle, where no comparable in vivo melatonin-plus-cisplatin skeletal-muscle study or muscle-specific clinical study has been identified.

### 5.4. Dose, Route and Timing of Melatonin Exposure

Melatonin regimens differ so widely across the evidence classes that no dose, route or schedule can be recommended for protecting skeletal muscle during cisplatin therapy (Table 2). In the direct C2C12 study, 100 µM melatonin was applied as a 24 h pretreatment before a 24 h, 30 µM cisplatin challenge [15]; whether melatonin remained in the medium during the challenge is not reported. Direct evidence therefore concerns preconditioning only, and no post-injury arm has been tested in muscle-lineage cells.

Among the non-cisplatin skeletal-muscle models and the non-muscle cisplatin models, melatonin was given intraperitoneally or orally; oral delivery as food pellets, capsules, or drinking water is a delivery mode within that route, not a separate route. Representative animal doses ranged from approximately 0.5 to 30 mg/kg/day, an approximately 60-fold range. One dose-comparison study in the mouse ovary reported protection at 20 mg/kg but not at 5 or 10 mg/kg [58], an organ- and protocol-specific threshold, not an established optimal dose.

Timing has been compared directly only in the kidney. Melatonin given for 5 days after a single cisplatin dose reduced lipid peroxidation and renal injury markers, and a regimen begun 5 days before cisplatin and continued 5 days afterward gave more complete histological preservation [60]. This post-cisplatin-only arm is the sole precedent in this evidence base for melatonin given after cisplatin exposure; it shows modification of injury in a non-muscle organ, not therapeutic efficacy in skeletal muscle. In a fully reported human renal trial, 20 mg/day orally from 24 h before cisplatin through 5 days afterward did not significantly reduce acute kidney injury [63].

The evidence therefore cannot distinguish preventive from therapeutic use in skeletal muscle. Direct data are preventive; most indirect animal data are pretreatment or bracketing designs, and only the kidney study above supplies a post-exposure-only comparator, in a non-muscle organ. No validated skeletal-muscle dose exists, none has been established as optimal, and the 100 µM cell-culture concentration cannot be converted into a human-equivalent dose. Dose, route, timing, and duration for skeletal-muscle protection, if protection occurs, must be established experimentally (Section 6.3).

### 5.5. Antitumor-Compatibility Context

A strategy that protected muscle but blunted cisplatin’s anticancer activity would be clinically unacceptable, so antitumor compatibility is a separate question from muscle protection. The available evidence addresses it at three levels, and none of them isolates a cisplatin-specific interaction in patients.

In malignant cells, melatonin did not protect rhabdomyosarcoma cell lines or primary mouse rhabdomyosarcoma cultures from concurrent cisplatin and was associated with greater sensitivity to the drug [67]. In a gastric-cancer cell line, including a CD133-positive subpopulation, concurrent melatonin and cisplatin increased the apoptotic fraction and G0/G1 accumulation compared with either agent alone [68]. Neither study reported a formal interaction analysis, so the combination is not described here as synergistic. Melatonin alone also had antiproliferative or anti-stemness activity at the millimolar concentrations used, which may account for part of the combined effect. These findings apply to the malignant-cell systems tested and are not extended to other tumor types.

No tumor-bearing animal experiment combining melatonin and cisplatin was identified in the evidence assembled for this review.

Human evidence exists for concomitant use of melatonin with cisplatin-containing combination chemotherapy, but it does not isolate a cisplatin-specific interaction and does not test skeletal-muscle protection. In randomized trials in advanced non-small-cell lung cancer, oral melatonin was given alongside cisplatin–etoposide chemotherapy [69,70]. A larger randomized study in metastatic solid tumors added melatonin to several chemotherapy regimens, including cisplatin-containing ones [71]. These reports did not show diminished overall antitumor activity with melatonin. Their interpretation is limited in four ways. First, cisplatin was always combined with etoposide or pooled with other regimens, so no effect can be attributed to cisplatin specifically. Second, the two lung-cancer reports share investigators, institution and treatment framework, and whether they describe independent cohorts cannot be determined from the publications. Third, a meta-analysis of randomized melatonin trials in solid tumors, which included this literature, found that the trials were open-label, described randomization and allocation concealment poorly, and came predominantly from one investigator network [72]. Finally, the absence of apparent antitumor impairment in historical combination-chemotherapy trials does not establish that a melatonin regimen designed to protect muscle during modern cisplatin-based treatment would be oncologically safe. None of these trials was designed to assess skeletal-muscle protection.

### 5.6. Clinical Context

Among the clinical studies identified, the randomized, placebo-controlled trial by Del Fabbro et al. [73] was specifically designed around advanced cancer and cachexia and assessed outcomes including weight, fat-free mass and appetite. This trial was not designed around cisplatin, did not select a platinum-treated population, and did not measure a skeletal-muscle-specific mechanistic endpoint; it did not demonstrate benefit on the reported cachexia outcomes. It provides contextual human evidence but does not test the question posed here, and its null findings cannot be used to infer that melatonin is ineffective against cisplatin-specific muscle toxicity. The boundary that emerges from Section 5.1 to Section 5.6 is clear. Direct evidence is confined to cells, indirect evidence to other insults or other organs, and clinical evidence to different conditions or to antitumor compatibility. At this point, methodological requirements, rather than further evidence appraisal, determine what can be done next. Table 2 summarizes the melatonin evidence by class, and Figure 3 maps the boundary between direct and indirect evidence.

**Table 2:** Selected melatonin evidence relevant to the cisplatin skeletal-muscle question.

Source	Evidence Class	Model/Tissue	Cisplatin Exposure	Melatonin Exposure	Principal Relevant Finding	Evidential Contribution	What the Study Does Not Establish
Baldassarri et al. [15]	A	C2C12 proliferating/undifferentiated myoblasts, in vitro	30 µM, 24 h	100 µM, 24 h pretreatment before the cisplatin challenge	Melatonin pretreatment attenuated selected apoptotic/mitochondrial-injury endpoints (TEM, TUNEL/CLSM, cardiolipin/NAO); a caspase-9 inhibitor characterized cisplatin’s own injury pathway, not melatonin’s mechanism	Direct cell-level anchor for apoptosis/mitochondrial injury	No mass/fiber/function endpoint; no animal arm or receptor/autophagy test; high in vitro concentration; combination-arm flow-cytometry quantification uncertain
Baldassarri et al. [14] (supplement abstract)	A	C2C12 myoblasts and myotubes, in vitro	Among several triggers (H_2_O_2_, cisplatin, etoposide, staurosporine); dose/duration not reported	Pretreatment; dose/duration not reported	Abstract of record: melatonin pretreatment prevented apoptosis induced by H_2_O_2_, cisplatin and etoposide; staurosporine-induced apoptosis was not inhibited. TEM and TUNEL-CLSM; myoblast and myotube results not separated	Direct evidence includes myotubes, but supplement-abstract level only	Dose, duration, n, stats, and effect size unavailable; no mass/function; independence unresolved; staurosporine outcomes conflict
Salucci et al. [48]	C	Undifferentiated C2C12 myoblasts, in vitro	None (H_2_O_2_, etoposide, staurosporine)	Pretreatment	Melatonin attenuated oxidative stress and apoptosis in a chemically distinct, non-cisplatin injury model; mitochondrial pathway implicated	Non-cisplatin cytoprotection supports mitochondrial/redox plausibility	No cisplatin or in vivo atrophy endpoint; trigger-specific completeness unresolved
Hibaoui et al. [11]	C	Primary skeletal-muscle cultures, C57BL/6J mice	None (tert-butyl-hydroperoxide oxidative challenge)	Present	Melatonin attenuated mitochondrial depolarization and permeability-transition-pore opening; maintained a more reduced intracellular environment; protected against subsequent cell death	Muscle-cell mitochondrial/redox evidence under oxidative stress	Not chemotherapeutic; no cisplatin; no in vivo endpoint
Hibaoui et al. [12]	C	mdx5Cv mice (Duchenne dystrophy model), triceps surae	None (genetic dystrophy model)	In vivo treatment	Improved twitch/maximal tetanic tension; reduced plasma creatine kinase activity; improved muscle redox status	In vivo functional/biochemical evidence in dystrophy	Dystrophin defect not corrected; not acquired toxic injury; no cisplatin
Su et al. [13]	C	Glycerol-induced tibialis anterior injury (mouse) + C2C12 mechanistic arm	None (glycerol chemical injury)	In vivo treatment	Improved rotarod performance, structural readouts, and fiber-size measures during early recovery; linked to Pax7-dependent myogenic differentiation	In vivo regeneration/structure evidence in chemical injury	No cisplatin; cell and animal mechanisms not merged
Salagre et al. [49]	C	Zucker diabetic fatty rat, red vastus lateralis	None (chronic metabolic disease model)	In vivo treatment	Altered muscle structure and mitochondrial fission/fusion protein expression; changed oxidative phenotype and steady-state LC3/p62-related markers	Mitochondrial/structural evidence in chronic metabolic disease	Steady-state autophagy markers only; no flux; no cisplatin
Kilic et al. [61]	D	Rat kidney, cisplatin nephrotoxicity model	Present	Present, co-treatment	Reduced renal dysfunction/tubular damage; increased nuclear Nrf2 and cytosolic HO-1; reduced NF-κB/AP-1	Pathway-specific Nrf2/HO-1 kidney protection	Kidney-specific; no receptor-manipulation test; not transferred to muscle
Ghadrdan et al. [62]	D	Human, cancer patients receiving cisplatin (kidney, clinical)	Present (standard chemotherapy)	Oral melatonin; exact dose and timing not independently verified in this review	Numerically fewer AKI episodes and smaller changes in selected urinary injury biomarkers; small study, not statistically significant for AKI prevention	Human clinical cisplatin-nephrotoxicity context	No muscle endpoint; small study; AKI prevention not significant
Barberino et al. [58]	D	Mouse ovarian cisplatin model (non-muscle organ)	Present	Present, with nonselective MT1/MT2 and selective MT2 receptor antagonists	Nonselective antagonist inhibited melatonin-associated ovarian protection; selective MT2 antagonist did not—a pattern supporting MT1 involvement in the ovary	Receptor-dependent cisplatin protection demonstrated in ovary	Ovary-specific; does not establish MT1 dependence in muscle; no muscle tissue
Jang et al. [59]	D	Mouse ovarian cisplatin model (non-muscle organ)	Present	Present	Implicated the PTEN/AKT/FOXO3a axis in melatonin’s protective context in ovary	PTEN/AKT/FOXO3a overlap in ovary	Does not establish pathway dependence in muscle
Zhuo & Jiang [66]	D	Rat heart (cardiac), cisplatin cardiotoxicity model	Present	Present, with BDNF-receptor antagonist (ANA-12) arm	Attenuated cardiac troponin/LDH-1 elevation and apoptotic markers; BDNF antagonism abolished the effect	Pathway-antagonist evidence in cisplatin cardiotoxicity	Heart-specific; no muscle evidence
Del Fabbro et al. [73]	E	Randomized, placebo-controlled clinical trial; advanced-cancer/cachexia patients	Not platinum-specific; no platinum-selected population	Oral melatonin vs. placebo	Tested melatonin against cachexia-related outcomes (weight, fat-free mass, appetite); did not demonstrate benefit on the reported outcomes	Human cancer-cachexia context	Not platinum-specific or a cisplatin-myotoxicity trial; no muscle-specific mechanism; null cachexia results do not establish cisplatin-specific inefficacy
Codenotti et al. [67]	E	Rhabdomyosarcoma malignant-cell model	Present	Present	Melatonin did not shield malignant cells from cisplatin; associated with greater drug sensitivity	Antitumor-compatibility context in rhabdomyosarcoma cells	No verified interaction analysis or synergy claim; not generalizable beyond rhabdomyosarcoma; no patient data; not skeletal muscle
Cheng et al. [68]	E	Gastric-cancer/cancer-stem-cell model	Present	Present	Related sensitization pattern reported in a gastric-cancer/cancer-stem-cell context	Second malignant-cell compatibility context	No verified interaction analysis; not generalizable across tumors; no patient data; not muscle tissue
Yao et al. [56]	C	Mouse cecal ligation and puncture sepsis, tibialis anterior; LPS-treated C2C12 myotubes (separate)	None (sepsis/LPS)	i.p., 7-day pretreatment before CLP; 24 h pretreatment in cells; animal dose not stated	Melatonin pretreatment reported to attenuate sepsis-associated fiber CSA reduction, with MuRF1 and PI3K/Akt changes; Akt dependence tested with MK-2206 in C2C12 only	PI3K/Akt and MuRF1 marker evidence in inflammatory atrophy	No in vivo Akt-dependence, muscle mass/function, atrogin-1 response, post-injury efficacy, or animal dose
Potes et al. [57]	C	ob/ob leptin-deficient and WT mouse hindlimb muscle	None (metabolic obesity model)	500 µg/kg i.p. daily for 4 weeks	Melatonin modified muscle bioenergetics and protein-quality-control markers, including MuRF1, Beclin-1, LC3, p62 and Lamp2a	Proteostasis/autophagy-marker context in non-cisplatin muscle	No autophagic flux, cisplatin-specific effect, or direct function
Zhou et al. [45]	C	Naturally aged male C57BL/6J mice; gastrocnemius	None (aging model)	10 mg/kg/day for 16 weeks	Higher gastrocnemius mass ratio, larger fiber CSA and improved forelimb grip; gut–muscle-axis correlates reported	Structural and functional evidence in aging	No cisplatin; gut-muscle mechanism not transferred to toxic atrophy
Fernández-Martínez et al. [46]	C	iMS-Bmal1−/− mice, male and female	None (circadian-disruption model)	10 mg/kg/day in food pellets for 2 weeks	Melatonin-alone arm improved muscle mass/fiber-size measures, treadmill-related performance and selected mitochondrial/ultrastructural outcomes	Structural, functional, and mitochondrial evidence with both sexes	No cisplatin; not equivalent to acute toxic injury
Ramírez-Casas et al. [47]	C	iMS-Bmal1−/− mice, male; mechanistic follow-up	None (circadian-disruption model)	Melatonin-alone arm present; exact dose, route, timing and duration unresolved	Myogenic gene expression, mitochondrial dynamics and respirometry were modified; no de novo structural/functional phenotype was established	Mechanistic complement to the phenotype study	Exact regimen and cohort independence unresolved; no de novo mass/CSA/function
Lissoni et al. [69]	E	Randomized advanced NSCLC clinical trial	Cisplatin + etoposide	20 mg/day oral melatonin, concomitant	No evidence of diminished overall antitumor activity; 1-year survival signal reported	Human antitumor-compatibility context with cisplatin-containing chemotherapy	No cisplatin-specific interaction, muscle protection, or oncologic safety of a muscle-protective regimen
Lissoni et al. [70]	E	Randomized metastatic NSCLC clinical trial	Cisplatin + etoposide	20 mg/day oral melatonin, concomitant	Tumor-regression and long-term-survival signals reported	Human antitumor-compatibility context with cisplatin-containing chemotherapy	No cisplatin-specific interaction; cohort independence unresolved; no muscle protection

Evidence classes are organizational categories, not quality grades. A: direct melatonin + cisplatin + normal skeletal-muscle/myogenic-cell evidence; B: cisplatin skeletal-muscle evidence without melatonin, summarized separately in Section 2 and Section 3 and Table 1 and therefore not repeated here; C: melatonin skeletal-muscle evidence from non-cisplatin models; D: melatonin + cisplatin evidence from non-muscle organs; E: broader mechanistic/translational context, including malignant-cell and human antitumor-compatibility evidence. Classes C, D and E are indirect with respect to the central question and are never presented as direct proof. The two Baldassarri publication records [14,15] are from the same research group; their experimental independence is unresolved. Table 2 is representative rather than exhaustive.

## 6. Methodological Limitations, Translational Gaps and Research Priorities

### 6.1. Direct-Evidence Limitations

The direct evidence base for melatonin in cisplatin-exposed skeletal muscle consists of the two publication records described in Section 5.1: one full article in proliferating myoblasts and one supplement abstract that adds a myotube arm without usable quantitative detail. No verified in vivo melatonin-plus-cisplatin skeletal-muscle study was identified in the targeted searches through 22 September 2026, and no clinical study specifically testing melatonin for cisplatin-associated skeletal-muscle loss was identified. The full article contains no muscle-mass, fiber CSA or diameter, strength, or force endpoint; it used a high in vitro melatonin concentration (100 µM) and a 24 h pretreatment design rather than concurrent or post-exposure treatment, with no post-injury direct muscle-lineage arm. Quantitative depth is limited even within the available direct records, which report selected apoptotic and mitochondrial-injury endpoints rather than autophagy, regeneration, muscle structure, or function.

### 6.2. Limitations Inherited from the Cisplatin Muscle Field

Because any melatonin experiment will be built on the existing cisplatin skeletal-muscle literature, that literature’s design limitations become design requirements. Dose, schedule, and muscle selection vary widely across the tumor-free studies summarized in Section 2, and male and young animals predominate, with Huot et al. [7] supplying the principal age- and sex-stratified dataset in the representative set. The intake-related confounding described in Section 2 has a direct consequence here: because DR and pair-feeding comparators establish that selected muscle findings exceed intake restriction without excluding every indirect systemic effect of cisplatin, a melatonin experiment should address intake explicitly rather than relying on a vehicle control alone. The endpoint framework carries over as well. A study reporting only whole-muscle mass would inherit the organ-level limitation set out in Section 2, and the structure–function dissociations documented in Section 3 mean that structural preservation cannot be assumed to imply functional benefit. Any autophagy claim would face the flux problem set out in Section 3.4. Finally, tumor-bearing designs cannot be used to infer tumor-free drug effects, and that separation must be preserved if melatonin is eventually tested under tumor burden.

Although cisplatin-associated reductions in myofiber cross-sectional area are well documented, the available evidence has not quantitatively resolved whether radial atrophy reflects fewer myofibrils per fiber cross-section, smaller individual myofibrils, or both. Similarly, whether cisplatin alters muscle-fiber length—and, if so, whether this involves changes in sarcomere length and/or serial sarcomere number—remains unexamined in the verified evidence base.

### 6.3. The Missing In Vivo Experiment

The evidence gaps point to one well-defined experiment rather than an open-ended list of possible studies. A tumor-free cisplatin model would remove tumor burden as a confounder, although it would not isolate direct muscle toxicity from the other systemic effects of cisplatin. At minimum, the design should include vehicle, cisplatin-alone, melatonin-alone, and combined melatonin-plus-cisplatin arms, with a DR or pair-fed comparator where feasible. Melatonin timing relative to cisplatin dosing should be pre-specified, and dose and route should be justified from melatonin pharmacology rather than extrapolated to a human dose. Core outcomes should include whole-muscle mass, quantified fiber morphometry, and a direct functional measure such as grip strength or contractile force, paired with molecular endpoints selected to match the mechanistic domains already implicated in cisplatin-exposed muscle, such as proteostasis and mitochondrial/redox markers. If an in vitro mechanistic component is included, a differentiated-myotube arm would extend beyond the myoblast-restricted direct evidence currently available, and animal and cell results should be reported and interpreted separately. This is a coherent endpoint framework, not an exhaustive panel; a single well-controlled study addressing structure, function, and matched mechanism would directly address the central in vivo evidence gap.

### 6.4. Receptor and Mechanism Testing

Any claim that a melatonin effect in cisplatin-exposed muscle depends on MT1 or MT2 signaling would require a receptor-antagonist or genetic strategy applied in skeletal muscle itself, on the model of the ovarian design discussed in Section 5.3, rather than an assumption carried over from a different organ. A claim about autophagy would require a dynamic flux design. More broadly, any pathway proposed as causally responsible for a melatonin effect would need an experiment in which manipulating that pathway changes the relevant outcome. This does not mean that every mechanistic domain identified in Section 3 must be tested within a single first animal study; a staged approach beginning with the core structural and functional endpoints is a reasonable priority.

### 6.5. Antitumor Compatibility and Translation

**Figure 4:**
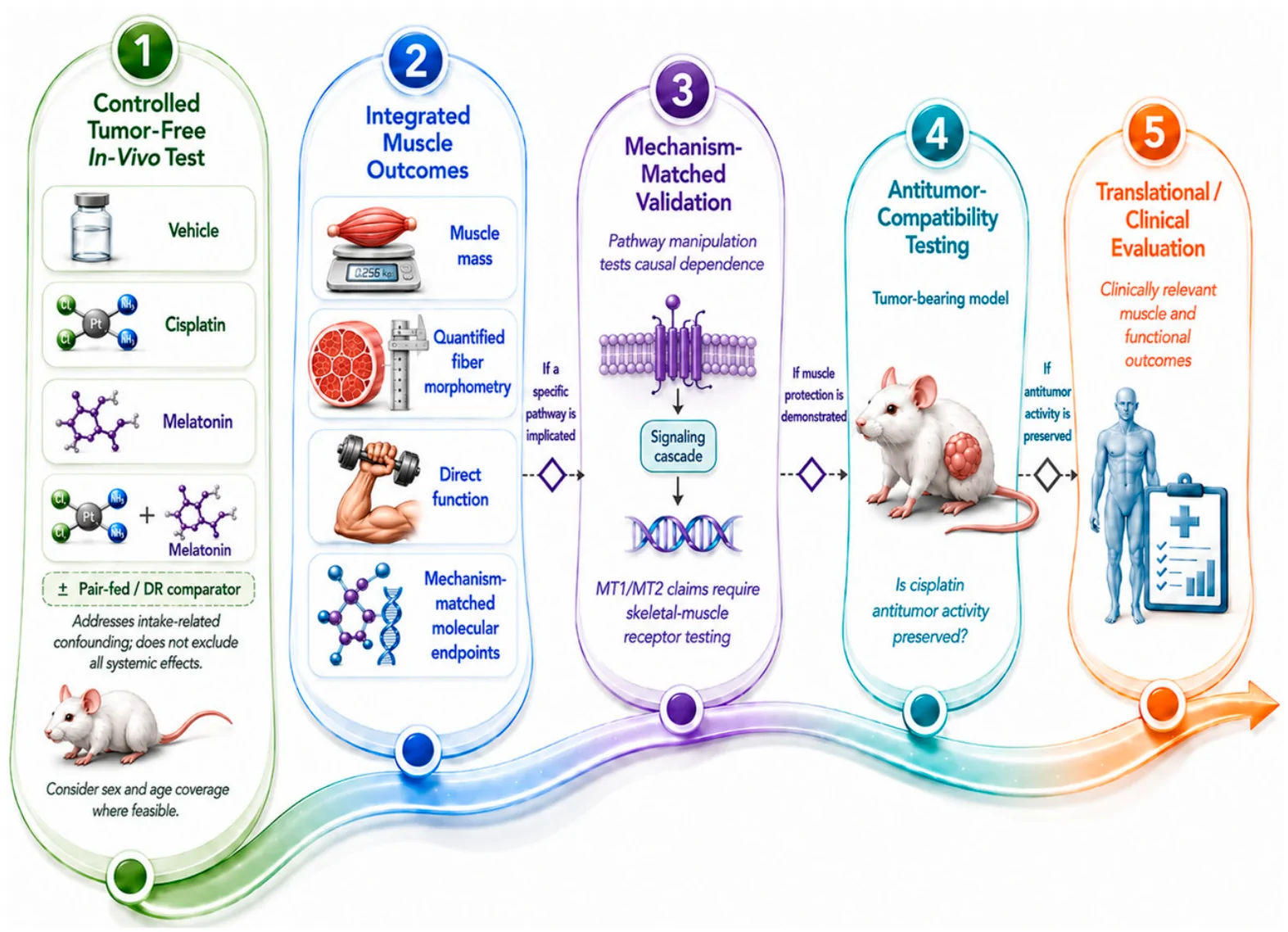
Translational roadmap and minimal design requirements for the missing in vivo melatonin-plus-cisplatin skeletal-muscle study. This conceptual, staged roadmap begins with a controlled tumor-free in vivo test (vehicle, cisplatin, melatonin, and combined arms, with an optional pair-fed or dietary-restriction (DR) comparator that addresses intake-related confounding without excluding all systemic effects of cisplatin), assessed through integrated outcomes that keep muscle mass, quantified fiber morphometry, direct function, and mechanism-matched molecular endpoints distinct. Subsequent stages are explicitly conditional: pathway-manipulation experiments that test, rather than assume, causal dependence for the specific mechanism proposed, with MT1 or MT2 receptor dependence, if specifically claimed, requiring receptor-antagonist or genetic testing performed in skeletal muscle itself; tumor-bearing antitumor-compatibility testing once muscle protection is demonstrated; and translational or clinical evaluation only if antitumor activity is preserved. No stage in this figure represents completed data or implies current clinical efficacy.

Future in vivo work should keep supportive muscle protection conceptually and experimentally separate from cisplatin’s antitumor efficacy. A controlled tumor-free experiment can test whether melatonin protects skeletal muscle under a more interpretable drug-exposure design, but translational validation then requires a tumor-bearing study that asks two distinct questions: whether muscle protection persists under tumor burden and whether cisplatin’s antitumor activity is preserved. The malignant-cell evidence and historical human trials of melatonin with cisplatin-containing combination chemotherapy discussed in Section 5.5 do not establish oncologic safety or a cisplatin-specific interaction, and they do not test skeletal-muscle protection. No clinical efficacy claim for melatonin against cisplatin-associated skeletal-muscle loss can currently be made. A translational roadmap and the minimal design requirements for the missing in vivo experiment are summarized in Figure 4.

## 7. Conclusions

Cisplatin produces a reproducible skeletal-muscle phenotype in tumor-free rodents, reducing muscle mass across several muscles, reducing myofiber size, and impairing strength, endurance, or contractile force. Tumor-bearing models show that this loss also occurs under tumor burden, but most lack a tumor-free cisplatin comparator, so drug and tumor effects remain only partly separable. The mechanistic evidence is multi-domain, implicating proteostasis, anabolic signaling, ER stress, mitochondrial and redox function, regulated cell death, and innate immune signaling. It is not, however, a settled causal account. Dose, schedule, muscle, and collection time vary widely; several interventions attenuate part of the phenotype and leave a measurable residue; and structural, functional, and molecular readouts dissociate often enough that no single pathway can be designated as the controlling lesion.

Melatonin engages several of the processes implicated in that phenotype. Experiments in skeletal muscle under oxidative, dystrophic, regenerative, metabolic, inflammatory, aging-related, and circadian conditions show that it can act at cellular and tissue levels. Direct evidence under cisplatin is limited to C2C12 cells, comes from one research group, uses a pretreatment design, and includes no structural or functional endpoint. No validated dose, route, or schedule exists for muscle protection. The available clinical data concern antitumor compatibility with cisplatin-containing combination regimens, not muscle outcomes. Biological plausibility is therefore supported, but protection of skeletal muscle has not been demonstrated.

The principal next step follows from that asymmetry: a well-controlled in vivo study in a tumor-free cisplatin model, with appropriately controlled arms and an endpoint set that integrates muscle mass, quantified morphometry, direct functional measurement, and mechanism-matched molecular readouts. Such a design removes tumor burden and tumor-response effects as contributors while retaining the systemic consequences of cisplatin exposure, and can test whether skeletal-muscle protection occurs in that setting. If that tumor-free study demonstrates protection, a separate tumor-bearing study is then required, not optional, to test whether the muscle-protective effect persists under tumor burden and whether cisplatin’s antitumor activity is preserved. Tumor-free and tumor-bearing findings answer different questions and are not substituted for one another. No clinical efficacy claim can be made from the evidence reviewed here.

## Data Availability

This study did not create or analyze new data. Data sharing does not apply to this article.
